# Association between hemoglobin glycation index and non-alcoholic fatty liver disease

**DOI:** 10.3389/fendo.2023.1094101

**Published:** 2023-02-07

**Authors:** Yuling Xing, Yunfeng Zhen, Liqun Yang, Lijing Huo, Huijuan Ma

**Affiliations:** ^1^ Department of Endocrinology, Hebei General Hospital, Shijiazhuang, China; ^2^ Department of School of Post Graduate Studies, Hebei Medical University, Shijiazhuang, Hebei, China; ^3^ Department of Clinical Laboratory, Hebei General Hospital, Shijiazhuang, China; ^4^ Hebei Key Laboratory of Metabolic Diseases, Hebei General Hospital Shijiazhuang, Hebei, China; ^5^ Department of Internal Medicine, Hebei Medical University, Shijiazhuang, Hebei, China

**Keywords:** hemoglobin glycation index, nonalcoholic fatty liver disease, body mass index, obesity, glycated hemoglobin A 1c

## Abstract

**Objective:**

The hemoglobin glycation index (HGI) reflects biological variability in hemoglobin A1c. Even so, studies on the relationship between HGI and non-alcoholic fatty liver disease (NAFLD) are limited. Therefore, this study aimed to explore the relationship between HGI and NAFLD. In addition, the study also aimed to provide new methods to identify patients with a high risk for the development of NAFLD.

**Methods:**

This was a retrospective study based on physical examination data from Japan. Patients were divided into quartiles (Q1–Q4) according to their HGI level; the lowest quartile (Q1) was used as the reference group. Patents were also classified into two subgroups based on the presence or absence of NAFLD. Baseline characteristics between the groups were compared. Multivariate logistic regression analysis was used to investigate the association between the HGI and NAFLD. A mediation analysis examined the mediation relationship between HGI and NAFLD. Subgroup analyses were performed to the reliability of the results.

**Results:**

A total of 14280 patients were eligible for inclusion in this study; 2515 had NAFLD. Patients in the NAFLD group had higher levels of HGI than patients in the non-NAFLD group. Increases in HGI correlated with an increased risk of NAFLD. After adjusting for confounding factors, the multivariate logistic regression analysis revealed that HGI was positively related to the prevalence of NAFLD. In addition, mediation analysis showed that body mass index (BMI) partly mediated the indirect impact of HGI on NAFLD preference. Subgroup analyses were performed according to age, sex, smoking status, and waist circumference. Our results indicated that HGI significantly correlated with NAFLD in patients with one of the following factors: age ≤60 years, BMI >28 kg/m^2^, female sex, a history of smoking, and abdominal obesity.

**Conclusions:**

HGI was an independent risk factor for NAFLD, and BMI partly mediated the association between HGI and NAFLD.

## 1 Introduction

Non-alcoholic fatty liver disease (NAFLD) is a pathological syndrome characterized by excessive fat accumulation in hepatocytes in the absence of excessive alcohol consumption or other definite factors, including non-alcoholic fatty liver, non-alcoholic steatohepatitis (NASH), cirrhosis, and hepatocellular carcinoma (1–3). Previous studies have shown that NAFLD correlates with metabolic disorders related to insulin resistance (IR), such as type 2 diabetes mellitus, obesity, and metabolic syndromes. Additionally, NAFLD predisposes patients to atherosclerosis (4–7). Previous studies have shown that NAFLD correlates with increased cardiovascular disease (CVD) risks,chronic kidney disease and cancer (8–10). Globally, NAFLD is a common cause of chronic liver disease. The incidence of NAFLD increases annually, and the global prevalence of NAFLD is ~ 30% (11). The prevalence of NAFLD, in Asians is expected to increase by 20-35% during the next decade (12). This rapid increase in the incidences of NAFLD will significantly impact healthcare and economic systems worldwide.

Hemoglobin A1c (HbA1c) is a diagnostic tool for diabetes and pre-diabetes, and it reflects the mean plasma glucose levels over the last 2–3 months. Recent studies suggest that blood glucose levels and interindividual variations contribute to HbA1c levels (13, 14). Consequently, patients with similar mean plasma glucose levels may have differing HbA1c levels. mean plasma glucose levels account for approximately 60%–80% of the variance in HbA1c levels (15). Based on these limitations, Hempe et al (16) developed a mathematical method in 2002, known as the hemoglobin glycation index (HGI), to quantify the difference between measured and predicted HbA1c levels. Subsequent studies demonstrated that HGI correlates with the observed variabilities in HbA1c levels (17, 18). An assessment of HGI in the Actions to Control Cardiovascular Risk in Diabetes trial showed that patients with diabetes and a high HGI had increased risks for diabetic retinopathies and nephropathies. Similar findings were reported in the Diabetes Control and Complication Trial (19, 20). Previous studies have also demonstrated an association between HGI and the risk of CVD in type 2 diabetes mellitus patients (21–24). High HGI levels, even in non-diabetic patients, correlate with an increased risk for atherosclerosis (25, 26). Mi et al. (27) observed that HGI is an independent risk factor for the development of hypertension despite HbA1c levels. In another study, HGI positively correlated with an increased risk for CVD, independent of other glycemic control factors (26). In addition, a prospective cohort study in Korea showed that HGI correlates with significant morbidity in CVD in the presence of controlled HbA1c levels (23). These findings strongly suggest that HGI directly impacts CVD compared to other glucose‐controlling indexes.

Nevertheless, previous studies on HGI primarily focused on vascular complications associated with diabetes. As such, studies demonstrating a relationship between HGI and NAFLD are limited. Therefore, this study aimed to elucidate the association between HGI and NAFLD.

## 2 Materials and methods

### 2.1 Study design and the subjects

#### 2.1.1 Data sources

Data for this study were derived from the “Ectopic fat obesity presents the greatest risk for incident type 2 diabetes: a population-based longitudinal study” research (28). Patient data for this study were collected between 2004 and 2015. The ethics committee of Murakami Memorial Hospital approved the study. In addition, written informed consent was obtained from each patient for the inclusion of their data in this study. The authors of the original study waived copyright and related ownership for these data. Therefore, we used these data for secondary analysis without infringing on the rights of the authors.

#### 2.1.2 Inclusion and exclusion criteria

All patients who visited the Murakami Memorial Hospital for routine physical examinations between 2004 and 2015 were enrolled in this study. Patient data, inclusive of personal data and test indicators, were recorded. Patients with alcoholic liver disease, viral hepatitis, diabetes mellitus (including type 1 and type 2 diabetes, gestational diabetes, and special types of diabetes), fasting plasma glucose (FPG) ≥6.1 mmol/l HbA1c ≥6.5%, taking any medications at baseline, incomplete data, or excessive alcohol consumption (>  210 g per week in men and  >  140 g per week in women during the past 12 months) were excluded from this study.

#### 2.1.3 Data collection and measures

The following subject variables were included in the dataset: sex, age, smoking history, body mass index (BMI), waist circumference (WC), total cholesterol (TC), triglyceride (TG), high-density lipoprotein cholesterol (HDL-C), FPG, HbA1c, Aspartate aminotransferase (AST), alanine aminotransferase (ALT), γ-glutamyl transferase (GGT), systolic blood pressure (SBP), and diastolic blood pressure (DBP). In addition, a fatty liver was diagnosed through abdominal ultrasonography, performed by trained technicians.

### 2.2 Research methods

#### 2.2.1 Calculation of HGI

**Figure 1:**
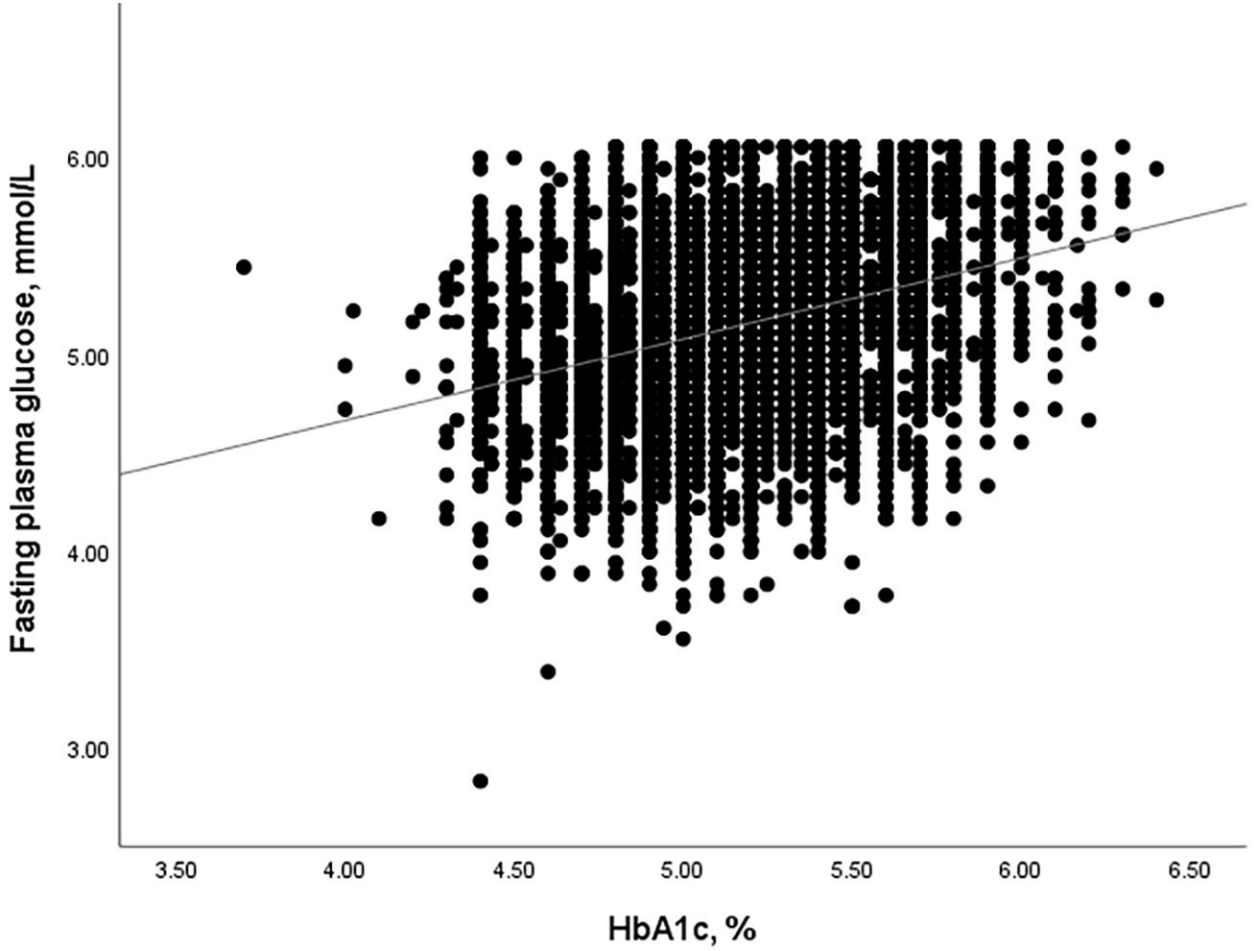
The correlation between HbA1c and HGI levels.

HGI was defined as the difference between the measured HbA1c levels and the predicted HbA1c levels (HGI = measured HbA1c - predicted HbA1c). Linear regression analysis was used to calculate the predicted HGI values (predicted HbA1c = 0.250 × FPG (mmol/L) + 3.889, r=0.321, and P<0.001, **Figure 1**).

#### 2.2.2 Statistical methods

Statistical analyses were performed with SPSS 25.0 statistical software. The results were expressed as the mean ± SD if the quantitative data were normally distributed. An independent sample t-test was used for intergroup comparisons, and multiple group comparisons were made with a one-way analysis of variance. Quantitative data that did not conform to the normal distribution are presented as the median (P25 and P75). The non-parametric Kruskal-Wallis test was used for comparisons between the different groups. Counting data were expressed as n (%), and a chi-square test was adopted. Pearson correlations were used for normally distributed data, while Spearman correlations were used for abnormally distributed data. Multivariate logistic regressions were performed to assess the associations between HGI and NAFLD. Subgroup analyses were performed to assess the robustness of the results in relation to specific variables.A likelihood ratio test was used to examine the interaction between TyG index and variables used for stratification. Finally, mediation analysis investigated factors that mediated the relationship between HGI and NAFLD. Differences were considered statistically significant at p < 0.05.

## 3 Results

### 3.1 Comparison of baseline characteristics between NAFLD and non-NAFLD groups

A total of 14280 patients were included in this study, including 7440 males and 6840 women, with an average age of 43.53 ( ± 8.89) years old; 11765 patients did not have NAFLD (non-NAFLD group), while 2515 patients had NAFLD (NAFLD group). At presentation, patients in the NAFLD group had significantly higher HGI, age, BMI, WC, TG, TC, AST, ALT, GGT, SBP, DBP, and lower HDL-C levels than patients in the non-NAFLD group. In addition, the number of smokers in the NAFLD group was higher than those in the non-NAFLD group (**Table 1**).

**Table 1:** Comparison of clinical indicators between the NAFLD and no NAFLD groups.

	All	NAFLD	non NAFLD	*P*
n	14280	2515	11765	
Sex(men)	7440(52.1%)	2037(81.0%)	5403(45.9%)	<0.001
Smoking	5529(38.7%)	1329(52.8%)	4200(35.7%)	<0.001
Age (years)	43.5326 ± 8.89083	44.7789 ± 8.32215	43.2662 ± 8.98568	<0.001
BMI (kg/m^2^)	22.0679 ± 3.13664	25.4988 ± 3.12574	21.3344 ± 2.60755	<0.001
WC(cm)	76.1961 ± 9.10003	85.983 ± 7.77706	74.1039 ± 7.9205	<0.001
ALT (IU/L)	19.7696 ± 14.45917	32.336 ± 19.35155	17.0833 ± 11.5221	<0.001
AST (IU/L)	18.227 ± 8.6625	22.3427 ± 9.78168	17.3471 ± 8.13869	<0.001
GGT (IU/L)	19.1538 ± 16.16541	28.4803 ± 22.08355	17.1601 ± 13.79843	<0.001
HDL-C (mmol/L)	1.4588 ± 0.40166	1.1838 ± 0.28988	1.5176 ± 0.39778	<0.001
TC (mmol/L)	5.1237 ± 0.86799	5.4418 ± 0.86749	5.0556 ± 0.85287	<0.001
TG (mmol/L)	0.8923 ± 0.63306	1.4355 ± 0.82706	0.7761 ± 0.51349	<0.001
HbA1c (%)	5.1778 ± 0.32094	5.3011 ± 0.33369	5.1514 ± 0.3119	<0.001
FPG (mmol/L)	5.1481 ± 0.41178	5.3951 ± 0.36373	5.0953 ± 0.40213	<0.001
SBP (mmHg)	113.9606 ± 14.83298	123.4425 ± 14.83352	111.9337 ± 14.02507	<0.001
DBP (mmHg)	71.1413 ± 10.39138	77.8312 ± 10.19138	69.7112 ± 9.86187	<0.001
HGI	0.0018 ± 0.30394	0.0634 ± 0.3156	-0.0114 ± 0.29977	<0.001

NAFLD, nonalcoholic fatty liver disease; BMI, body mass index; WC, waist circumference; ALT, alanine aminotransferase; AST, aspartate aminotranferase; GGT, γ-Glutamyl transferase; HDL-C, high density lipoprotein cholesterol; TC, total cholesterol; TG, triglyceride; HbA1c, hemoglobin A1c; FPG, fasting plasma glucose; SBP, systolic blood pressure; DBP, diastolic blood pressure; HGI, hemoglobin glycation index.

### 3.2 Baseline characteristics of study subjects according to the levels of HGI

**Figure 2:**
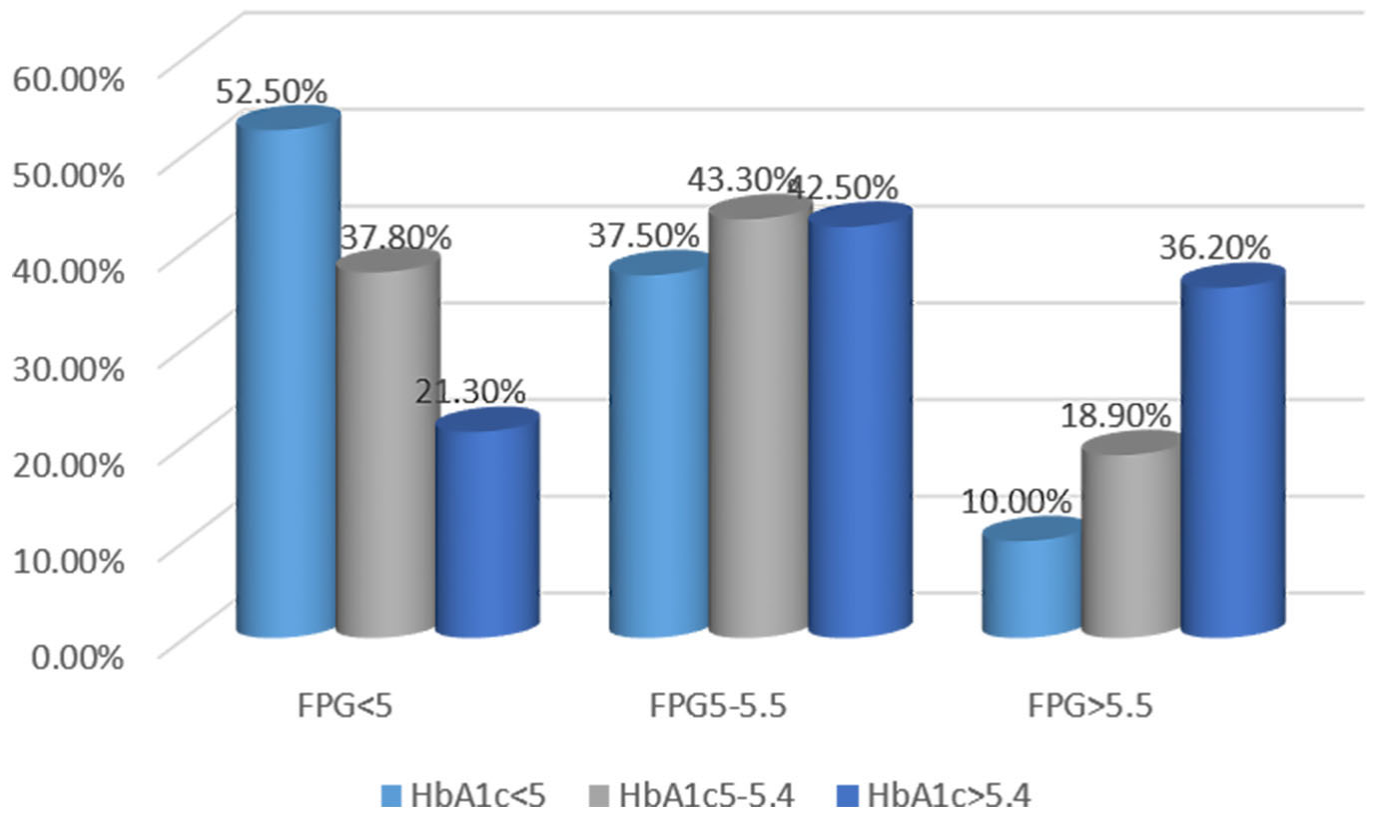
Distribution of HbA1c value at different FPG levels.

This study showed that 21.3% of patients had HbA1c levels > 5.4%, even in patients with FPG <5 mmol/L. HbA1c levels < 5% were identified in 10% of patients subjects with FPG >5.5 mmol/L. These results indirectly illustrate the need for further study on HGI (**Figure 2**).

Subjects were grouped into four groups (Q1-Q4) in ascending order according to the interquartile range of the HGI. There were proportional increases between HGI and TC levels,HGI and the female proportion, while the number of smokers proportionally decreased. Statistical differences were noted between the groups. The age of patients in the Q4 group was significantly higher than that of patients in the Q1, Q2, and Q3 groups. Similarly, the age of patients in the Q3 group was higher than those in the Q2 and Q1 groups. WC and BMI were higher in the Q4 group than in the other groups (Q1–3). The Q4 group had higher ALT and SBP levels than the Q2 and Q3 groups. AST levels in the Q4 group were higher than in the Q1, Q2, and Q3 groups. However, AST levels in the Q3 group were higher than in the Q1 group. HDL-C levels in the Q2, Q3, and Q4 groups were higher than in the Q1 group. TG levels in the Q4 group were significantly higher than in the Q2 group. Similarly, TG levels in the Q2 group were higher than in the Q1 group. Finally, DBP levels in the Q4 group were higher than in the Q3 group (**Table 2**).

**Table 2:** Clinical Characteristics of subjects by HGI Category.

	Q1	Q2	Q3	Q4	*P*
n	3571	3623	3501	3585	
Sex(men)	2224(29.9%)	1970(26.5%)	1742(23.4%)	1504(20.2%)	<0.001
Age (years)	42.159 ± 8.284	42.407 ± 8.475	43.647 ± 8.922	45.927 ± 9.346	<0.001
BMI (kg/m^2^)	22.007 ± 2.880	21.893 ± 3.032	21.982 ± 3.131	22.390 ± 3.454	<0.001
WC(cm)	76.123 ± 8.846	75.680 ± 8.873	75.968 ± 9.041	77.014 ± 9.570	<0.001
ALT (IU/L)	19.717 ± 17.830	19.343 ± 11.957	19.455 ± 12.474	20.560 ± 14.797	0.001
AST (IU/L)	17.511 ± 11.458	18.005 ± 6.867	18.111 ± 7.266	19.277 ± 8.203	<0.001
GGT (IU/L)	19.336 ± 16.984	19.085 ± 17.228	18.791 ± 14.197	19.397 ± 16.024	0.377
HDL-C (mmol/L)	1.396 ± 0.371	1.474 ± 0.397	1.491 ± 0.409	1.476 ± 0.421	<0.001
TC (mmol/L)	4.969 ± 0.813	5.057 ± 0.8449	5.146 ± 0.868	5.323 ± 0.904	<0.001
TG (mmol/L)	0.903 ± 0.617	0.854 ± 0.600	0.889 ± 0.684	0.924 ± 0.628	<0.001
HbA1c (%)	4.800 ± 0.178	5.068 ± 0.105	5.273 ± 0.136	5.572 ± 0.175	<0.001
FPG (mmol/L)	5.170 ± 0.398	5.114 ± 0.379	5.136 ± 0.453	5.172 ± 0.412	<0.001
SBP (mmHg)	114.119 ± 14.881	113.451 ± 14.435	113.651 ± 14.856	114.620 ± 15.135	0.004
DBP (mmHg)	71.557 ± 10.268	70.900 ± 10.118	70.685 ± 10.650	71.417 ± 10.510	0.001
HGI	-0.382 ± 0.152	-0.100 ± 0.0556	0.100 ± 0.063	0.390 ± 0.144	<0.001

**Figure 3:**
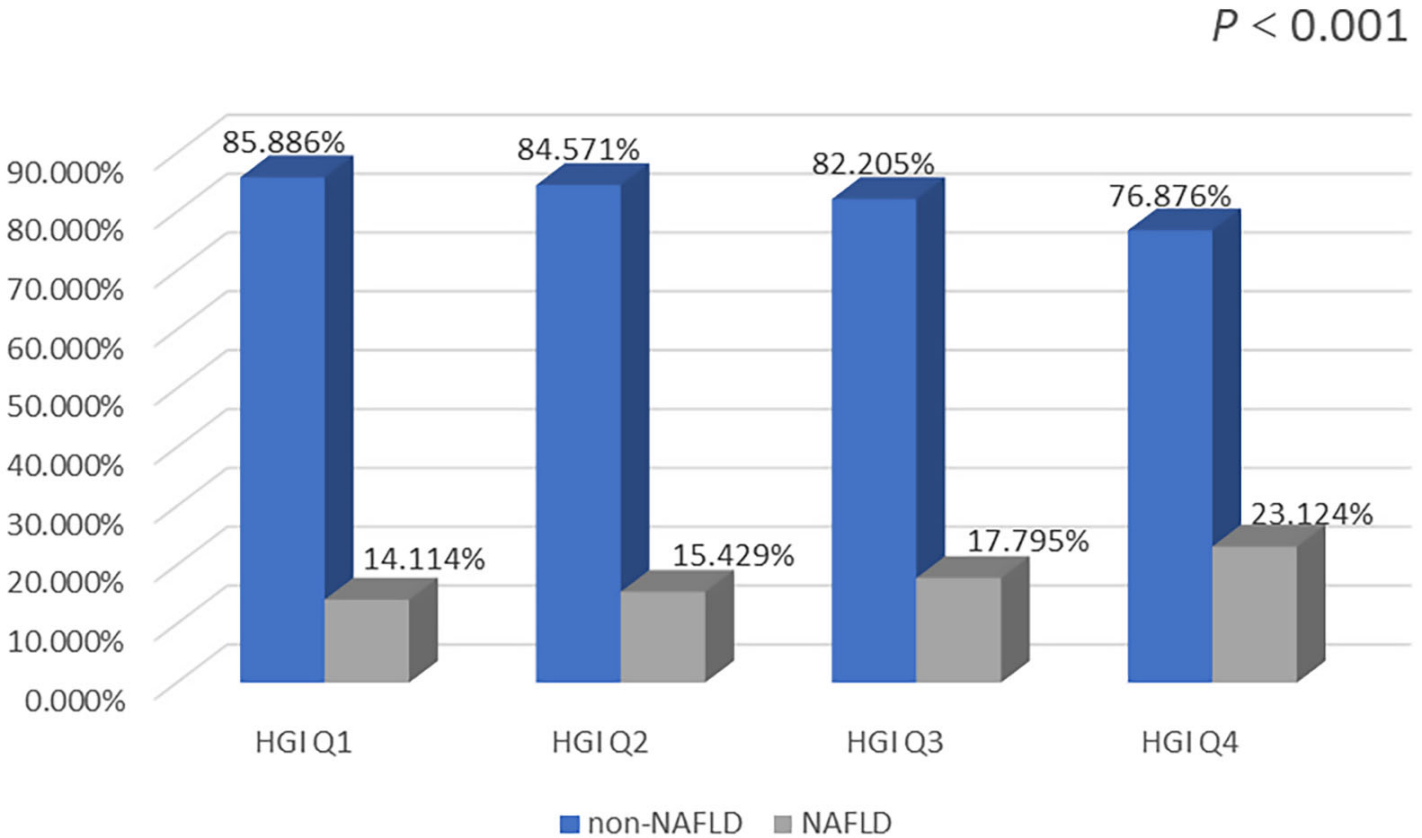
Comparison of NAFLD prevalence at different HGI levels.

The prevalence of NAFLD was markedly higher in the Q2, Q3, and Q4 groups than in the Q1 group. In addition, the Q4 group had the highest prevalence of NAFLD. The prevalence of NAFLD increased significantly with increasing HGI (**Figure 3**).

### 3.3 The correlations between HGI and potential risk factors of NAFLD

**Figure 4:**
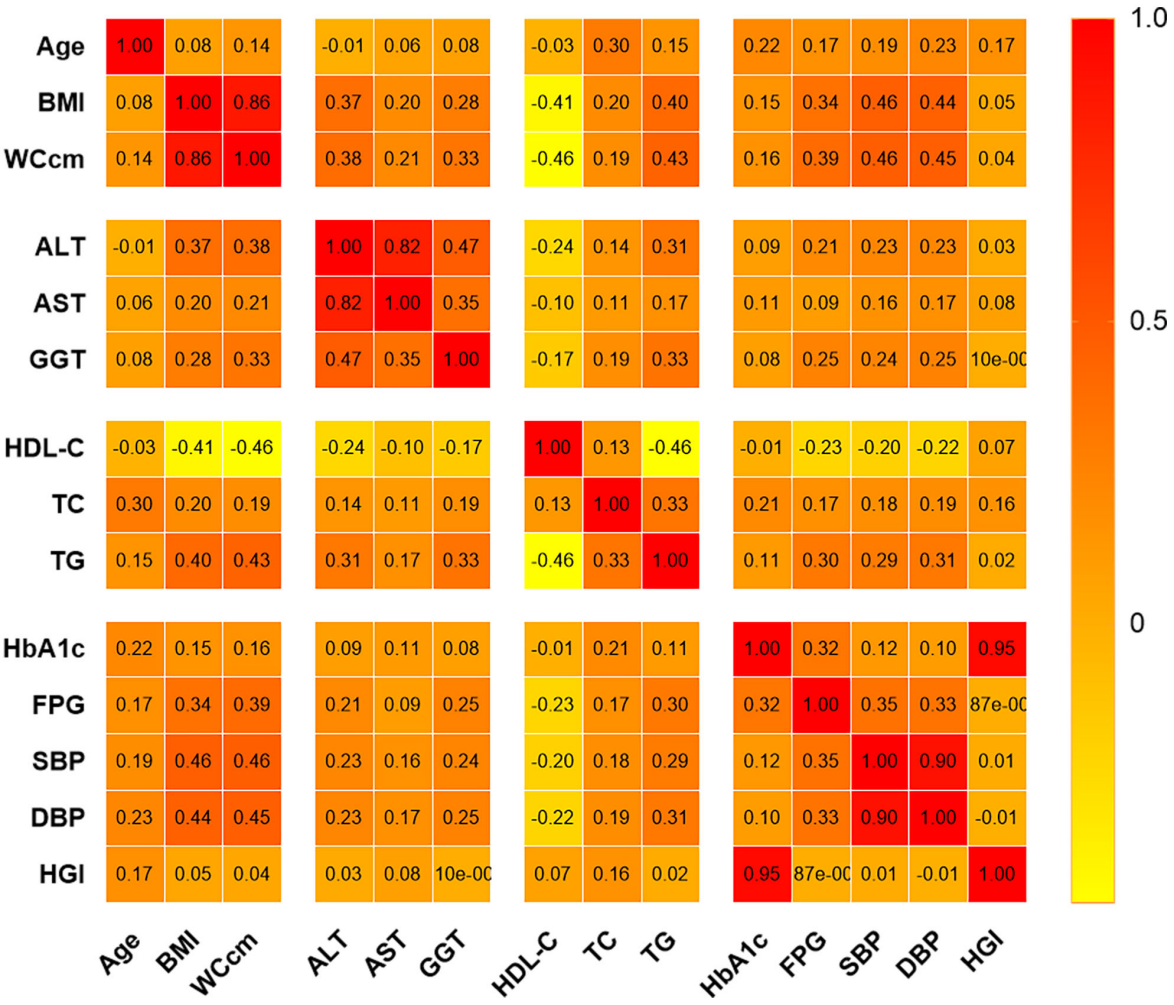
Correlation of HGI with potential NAFLD risk factors.

There were no significant correlations between HGI and GGT, DBP, or SBP. However, correlation analysis revealed a positive correlation between HGI and age (r=0.172, P<0.001), BMI (r=0.046, P<0.001), WC (r=0.038, P<0.001), ALT (r=0.025, P=0.003), AST (r=0.083, P<0.001), TC (r=0.161, P<0.001), HDL-C (r=0.071, P<0.001), and TG (r=0.017, P=0.045) (**Figure 4**).

### 3.4 The association between HGI and NAFLD in multivariable logistic regression analyses

Multivariate logistic regression analysis showed that HGI was an independent risk factor for NAFLD (OR 2.811; 95% CI, 2.313-3.417; P < 0.001) after adjusting for sex, age, smoking, BMI, WC, ALT, AST, GGT, HDL-C, TC, TG, SBP, and DBP. In addition, HGI was analyzed as a categorical variable to ensure the robustness of the results. The results showed a significant positive correlation between HGI and the risk of NAFLD (**Table 3**).

**Table 3:** Multivariate Logistic Regression Analysis of HGI for NAFLD.

Outcomes	Model I		Model II		Model III	
	OR (95% CI)	*P*	OR (95% CI)	*P*	OR (95% CI)	*P*
HGI	2.263(1.961,2.613)	<0.001	2.825(2.359,3.383)	<0.001	2.811(2.313,3.417)	<0.001
HGI (quartile)						
Q1	Ref		Ref		Ref	
Q2	1.11(0.974,1.265)	0.116	1.251(1.072,1.459)	0.004	1.347(1.139,1.592)	<0.001
Q3	1.317(1.159,1.497)	<0.001	1.542(1.323,1.797)	<0.001	1.583(1.34,1.87)	<0.001
Q4	1.83(1.62,2.068)	<0.001	2.197(1.887,2.556)	<0.001	2.184(1.85,2.578)	<0.001
*P* for trend	<0.001		<0.001		<0.001	

Model I adjusted for age,sex,smoking; Model II: further adjusted for WC,ALT,AST,regular exercise,GGT; Model III further adjusted for HDL,TG,TC,SBP,DBP.

### 3.5 Mediated effect of BMI on the association between HGI and NAFLD

**Figure 5:**
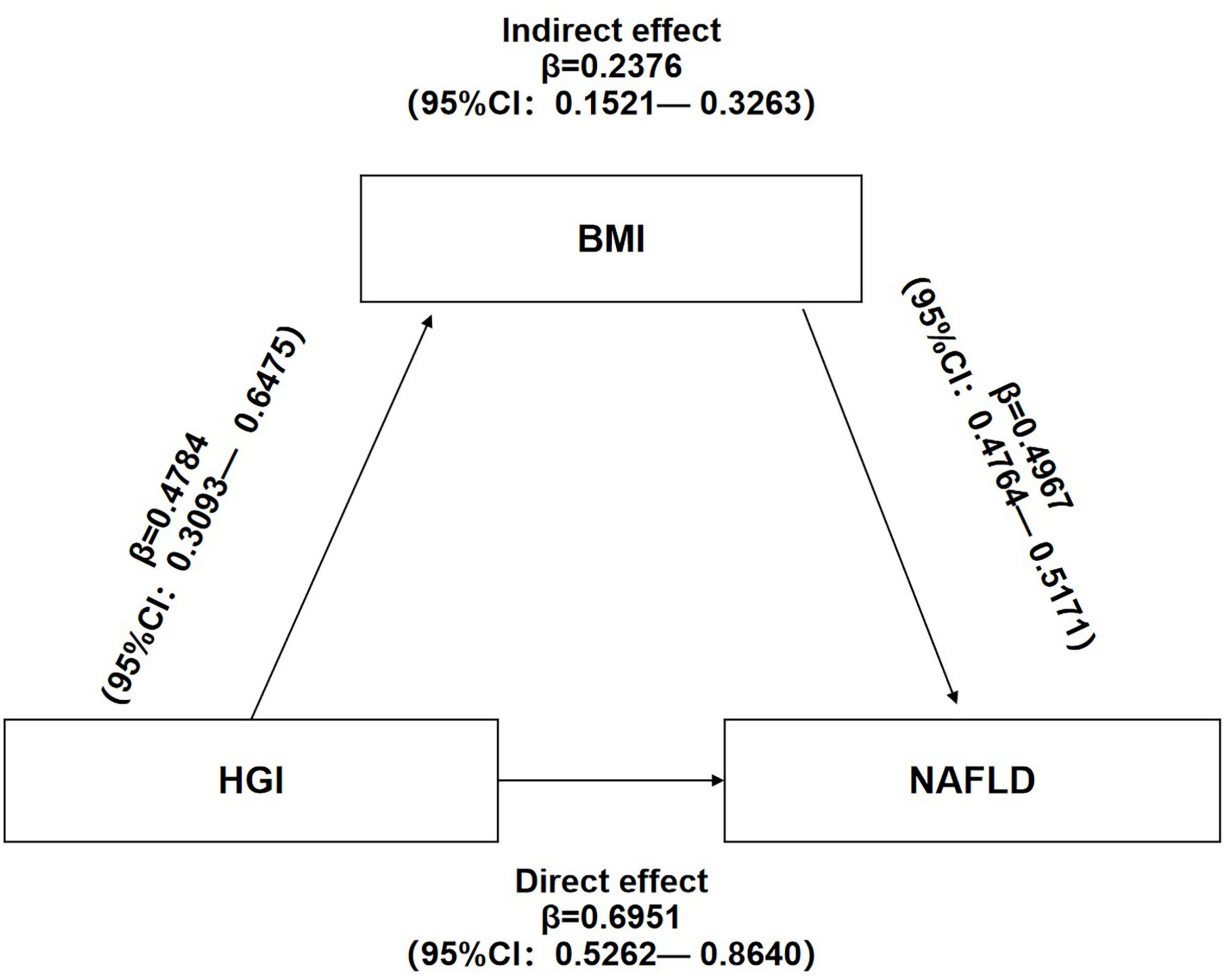
Mediation of BMI on the association between HGI and NAFLD.

Mediation analysis of the relationship between HGI, BMI, and NAFLD showed that HGI and BMI were risk factors for NAFLD. Additionally, HGI positively correlated with BMI. These results suggest potential mechanisms in which BMI links HGI with NAFLD. Mediation analysis also showed that HGI significantly impacted the prevalence of NAFLD (β=0.6951,95%CI:0.5262-0.8640) and that BMI partly mediated the indirect impact of HGI on the incidence of NAFLD (β=0.2376,95%CI:0.1521-0.3263). A mediated percentage of 25.47% was observed in the model (**Figure 5**).

### 3.6 Subgroup analyses

A subgroup analysis on age, sex, smoking, WC, and BMI in the study population was performed to determine variations in the association between HGI and NAFLD in different populations. The association of HGI and risk of NAFLD was stronger among those with a age less than 40 years and BMI>28kg/m^2^.No other significant interaction was observed in subgroup analyses. (**Table 4**).

**Table 4:** Subgroup analysis and interaction of the association of HGI with NAFLD.

Characteristics	No	OR (95%CI)	*P*	*P* for interaction
Age(years)				0.005
≤40	6215	3.707(2.613,5.260)	<0.001	
40-60	7578	2.811(2.313,3.417)	<0.001	
>60	487	1.058(0.393,2.849)	0.911	
BMI(kg/m2)				0.023
<23	9394	3.022(2.159,4.230)	<0.001	
23-25	2698	2.811(2.313,3.417)	<0.001	
25-28	1634	2.811(2.313,3.417)	<0.001	
>28	644	5.524(2.622,11.64)	<0.001	
Sex				0.156
Men	7440	2.619(2.090,3.281)	<0.001	
Women	6840	3.287(2.210,4.891)	<0.001	
visceral obesity				0.476
No	12424	2.584(2.067,3.231)	<0.001	
Yes	1856	3.577(2.376,5.384)	0.037	
Smoking				0.750
No-smoking	8751	2.588(1.953,3.431)	<0.001	
Ex-smoker	2672	2.556(1.725,3.789)	0.001	
Current-smoker	2957	3.529(2.418,5.15)	<0.001	

## 4 Discussion

In this study, the physical examination population was evaluated. Higher HGI levels were observed in the NAFLD group than in the non-NAFLD group. Furthermore, HGI increased simultaneously with the prevalence of NAFLD. Multivariate logistic regression analysis showed that HGI was an independent risk factor for NAFLD. Further analysis showed that patients in the highest quartile of HGI had significantly higher risks for NAFLD than those in the three lower quartile groups. A study conducted by Fiorentino et al. (29) demonstrated that HGI correlated with hepatic steatosis; this finding was congruent with the findings of this study. Our results are similar to these two studies (30, 31).,elevated HGI levels are independently associated with NAFLD.We also found that BMI partly mediates the association between HGI and NAFLD.Our research demonstrates several new populations that may be at risk for NAFLD including people younger than 40 with high HGI and obese people with high HGI,which were not previously described.

Several mechanisms may explain the association between HGI and NAFLD. First, intracellular glucose levels were higher in patients with higher HGIs than those with lower HGIs. Increased intracellular glucose release a significant number of noxious metabolites. This release ultimately leads to liver injury (18). Chronic inflammation is considered a core pathogenesis of the development of NAFLD (32, 33). Fiorentino et al. (29) also observed that there is a significant increase in the levels of inflammation-related biomarkers in patients with higher HGIs.

Similarly, Hu et al. (30) reported a positive correlation between HGI and inflammatory biomarkers, including WBC and platelet counts. The US National Health and Nutrition Examination Survey results suggest that HGI independently associates with inflammatory biomarkers (such as C-reactive protein, polymorphonuclear leukocytes, and monocytes.) (34) Inflammation might impair insulin signaling and exacerbate fatty liver infiltration. Alternatively, inflammation induces oxidative stress, damages mitochondria, and causes ER stress. Defective mitochondria are associated with incomplete fat oxidation and the generation of toxic lipid intermediates. These toxic lipid intermediates generate many reactive oxygen species, which eventually promote the progression of liver steatosis into more severe forms of the disease (35, 36) and fuel the transition from NAFLD to non-alcoholic steatohepatitis, liver cirrhosis, and even hepatocellular carcinoma (35, 37, 38). Previous studies reported that HGI represents the degree of non-enzymatic hemoglobin glycation (26, 29, 39). Non-enzymatic hemoglobin glycation might play a key role in NAFLD pathogenesis (40, 41). Studies have also shown that HGI reflects the level of advanced glycation end products (AGEs) (42). AGEs may alter the structure of proteins and their functions, resulting in alterations that contribute to histopathological changes in the liver (42). Studies have also demonstrated that AGEs, in combination with the activation of the RAGE downstream pathways, trigger further inflammation and oxidative stress and impair insulin signaling. Therefore, AGEs increase the development and progression of NAFLD (43). IR is recognized as a potential pathogenesis of NAFLD. In addition, HGI may associate with IR. Furthermore, the degree of IR in patients with lower HGIs was less than not those in patients with higher HGIs (26).

As a result of obesity, an imbalance in pro- and anti-inflammatory adipokines is secreted from adipose tissue. This secretion contributes to the development of NAFLD (44). Similarly, a previous investigation indicated that several adipokines, including adiponectin and leptin, play a role in the pathogenesis of NAFLD, including hepatic fat accumulation, chronic inflammation, and IR (45, 46). BMI is an easy-to-implement body composition classification screening indicator. BMI classifies patients as underweight, normal weight, overweight, or obesity. BMI is widely employed as a surrogate indicator for measuring weight status (adjusted for height) and the percentage of fat mass (47–49). Among anthropometric measurements, BMI has the best discriminatory power (50). Therefore, BMI is the most commonly used anthropometric measure of obesity (51, 52). Numerous studies have shown a strong correlation between BMI and NAFLD; the higher the BMI, the higher the risk of developing NAFLD (53–55). Tang Z et al. (56) noted that increases in BMI correlated with NAFLD and that these increases were independent of the patient’s age. The study also revealed that BMI modulates the association between physical examinations and blood biochemistry parameters and NAFLD (56).Obesity-related inflammatory input has a well-established correlation with NAFLD (22, 57). BMI is a poor predictor of NAFLD severity (58). Even so, BMI was confirmed as the most useful predictive factor for NAFLD onset in both sexes (59). BMI had the highest area under the curve in the prediction of NAFLD (60). BMI is superior to WC in predicting the risk for NAFLD (61). A recent study showed a significant correlation between HGI and obesity (26), which was consistent with the previous findings of Yoo et al. (31) Correspondingly, Hu et al. (30) found that HGI levels had positive associations with BMI. Therefore, a mediator analysis was conducted to determine if BMI mediates the correlation between HGI and NAFLD. Our study showed that HGI and BMI were positively associated with NAFLD, and that HGI positively correlated with BMI. The mediation analysis results indicated that the relationship between the HGI and NAFLD was partly mediated by obesity. In addition, the correlation between the HGI and NAFLD was significantly higher in obese patients.

This study identified that increases in HGI correlated with increased incidences in females. Previous studies also showed gender differences in NAFLD related to gender-specific gene expression, sex hormones, and gender-related economic, behavioral, and environmental factors (62, 63)..

This study had limitations. First, this was a cross-sectional study, and thus could not establish a cause-effect relationship. Further prospective studies are required to validate these findings. Second, HGI levels may vary by population (64, 65). Therefore, the results obtained from this study conducted in Japan might not be applicable to other ethnicities. Third, fatty liver diagnosed by ultrasonography may provide an incorrect diagnosis compared to a liver biopsy. Finally, since the dataset was obtained from a public database, it could not be updated. Therefore, we could not investigate the effect of HGI on liver fibrosis. Some known risk factors of demographic data for NAFLD, such as dietary preferences, exercise habits, work types, and so on, were not included, which limits a comprehensive assessment of the correlation between risk factors and NAFLD.

## 5 Conclusion

In conclusion, HGI positively correlates with the prevalence of NAFLD, and BMI partly mediates the association between HGI and NAFLD.Our research demonstrates several new populations that may be at risk for NAFLD including people younger than 40 with high HGI and obese people with high HGI.

## Data availability statement

The datasets presented in this study can be found in online repositories. The names of the repository/repositories and accession number(s) can be found below: https://doi.org/10.1038/s41366-018-0076-3.

## Ethics statement

The studies involving human participants were reviewed and approved by Murakami Memorial Hospital. The patients/participants provided their written informed consent to participate in this study.

## Author contributions

YX and YZ performed the data analysis and YX prepared the initial draft of the manuscript. LY and LH gave statistical advice throughout the data analyses and contributed to the manuscript. HM was responsible for the study concept, data analysis, and interpretation of the findings, and critically reviewed and revised the manuscript. All authors contributed to the article and approved the submitted version.
